# Cerebral Sinus and Venous Thrombosis Associated with von Willebrand Factor, Independently of Factor VIII

**DOI:** 10.4137/ccrep.s737

**Published:** 2008-05-14

**Authors:** Mari Terashima, Hiroshi Kataoka, Hirosei Horikawa, Hiroyuki Nakagawa, Toshiaki Taoka, Masanori Matsumoto, Kozue Saito, Kimihiko Kichikawa, Satoshi Ueno

**Affiliations:** From the Department of Neurology, Nara Medical University, Kashihara, Nara, Japan.; 1 The Department of Radiology, Nara Medical University, Kashihara, Nara, Japan.; 2 The Department of Blood Transfusion Medicine, Nara Medical University, Kashihara, Nara, Japan.

**Keywords:** von Willebrand factor, venous thrombosis, cerebral sinus, procoagulant factor VIII, rheolytic thrombectomy, venous infarction

## Abstract

**Background and purpose:**

Previous studies have linked procoagulant factor VIII (F VIII) to an increased risk of venous thrombosis, whereas the relation between plasma von Willebrand factor (VWF) and venous thrombosis remains poorly understood. Elevated VWF levels are frequently found in patients with cerebral sinus and venous thrombosis (CSVT), always in association with high F VIII levels. We describe a patient with CSVT accompanied by elevated VWF levels without high F VIII levels.

**Case description:**

A 23-year-old healthy man who had headache noticed difficulty in moving the right hand. On the following day, he lost consciousness and had partial seizures of the right hand. After regaining consciousness, weakness of the right extremities developed. The cranial angiogram confirmed occlusion of the superior sagittal sinus. The levels of VWF and F VIII were 238% and 101.9 IU/dl, respectively. We performed balloon percutaneous transluminal angioplasty and mechanical thrombectomy, leading to successful recanalization of the intracranial sinuses. VWF levels were decreased along with radiographic improvement, independently of F VIII.

**Conclusion:**

VWF may contribute to CSVT and that inhibition of VWF activity potentially has a role in the future treatment of pathological conditions related to venous thrombosis.

## Introduction

Von Willebrand factor (VWF) is an adhesive multimetric protein present in platelets, endothelial cells, and the subendothelium. VWF serves as a carrier for the procoagulant factor VIII (F VIII), protecting it from inactivation [1]. Some studies have shown a significantly increased risk of stroke in individuals with high VWF and F VIII levels [2]. High plasma concentrations of VWF have also been associated with an elevated risk of arterial thrombosis [3]. Previous studies have linked F VIII to an increased risk of venous thrombosis [4], whereas the relation between plasma VWF and venous thrombosis remains poorly understood, both in vitro and in vivo; whether a causal relation exists remains uncertain [5]. Here, we describe a patient with cerebral sinus and venous thrombosis (CSVT) accompanied by elevated VWF levels without high F VIII levels.

## Case Report

A 23-year-old healthy man with blood group A who had headache for 1 day, noticed difficulty in moving the right hand. On the following day, he lost consciousness and had partial seizures of the right hand. After regaining consciousness, weakness of the right extremities developed, and the patient was admitted to our hospital. On the day of admission, he was alert and fully oriented. The blood pressure was 132/63 mmHg. The results of general examinations were normal. The muscle strength of the right extremities was severely compromised. The deep tendon reflexes were normal, with no pathological reflexes. Cranial nerves and superficial sensation were not impaired. Blood cell counts and the results of routine biochemical analysis were normal, expect for an elevated creatine kinase level (1515 mg/dl). Brain MRI showed abnormally high signal intensity in the left frontal and parietal parenchyma on diffusion weighted, T2-weighted, and fluid-attenuated inversion recovery images. Gadolinium-enhanced T1-weighted images show attenuated enhancement in left superior sagittal sinus, and the left Rolandric and Troland veins were not detected, suggesting a venous thrombosis. Complementary screening studies for infectious, immunologic, hematologic, neoplastic, and systemic disorders, including tests for protein C, protein S, homocysteinaemia, lupus anticoagulant and anticardiolipin antibodies, showed no apparent cause of CSVT. There was no family or personal history of clotting disorders or the use of oral contraceptives. Ultrasound evaluation of the lower extremity veins found no evidence of thrombosis. He was given a diagnosis of CSVT and received intravenous heparin continuously and anticonvulsant treatments. Five days after admission, the level of consciousness decreased, and generalized convulsions developed. A cranial CT scan showed increased brain swelling, abnormally low signal intensities, and intracranial hemorrhage. Six days after admission, the initial angiogram confirmed occlusion of the superior sagittal sinus (SSS) (Fig. A). Transfemoral catheterization of the SSS was performed, and an Exelcior 1018 microcatheter was placed coaxially and advanced into the SSS. Because of cranial hemorrhage and the long time until catheter treatment, with a 140-cm Thrombuster II catheter, rheolytic thrombectomy was performed in the SSS (Fig. B). Subsequently, balloon percutaneous transluminal angioplasty (Gateway 3 × 9 mm balloon catheter) was done (Fig. D). For complete recanalization of the sinuses, we repeated balloon percutaneous transluminal angioplasty and mechanical thrombectomy. The final angiogram showed moderate recanalization of the SSS (Fig. C). Simultaneously, the level of consciousness dramatically increased. The patient became alert, but right hemiparesis persisted. The pathological diagnosis on aspiration of the thrombus was fibrin thrombosis without malignancy or inflammatory cells. After catheter therapy, intravenous heparin was administered. The levels of VWF and F VIII on day 8 were 238% and 101.9 IU/dl, respectively. The activity of a disintegrin and metalloproteinase with thrombospondin-1-like domain (ADAMTS-13) was normal. A cranial CT scan on day 18 showed the disappearance of brain swelling and hemorrhage and a decrease in abnormal low-signal intensities. VWF and F VIII levels on day 25 were 161% and 99 IU/dl, respectively. He regained strength in the right extremities and could walk unaided. Thirty-four days after admission, warfarin was started. Cranial MR venography on day 43 demonstrated dramatic recanalization of the SSS. The weakness of right extremities decreased further, and he required no assistance in activities of daily living. On day 56, the VWF level decreased to 131%. Conventional inflammatory markers such as C-reactive protein, were not elevated during the course. Sixty-days after admission, he left the hospital with no impairment of motor abilities or higher function.

## Discussion

We described a case of CSVT associated with high VWF levels. The increased VWF level in our patient decreased after recanalization of the venous sinus in response to anticoagulant therapy.

Few studies have examined the relationship between plasma VWF levels and venous thrombosis[6,7]. Elevated VWF levels are frequently found in patients with CSVT, nearly always in association with high F VIII levels[7]. The cutoff values for F VIII levels and VWF levels in patients with CSVT are 150 IU/dl and 150%, respectively, as compared with controls[7]. In our patient, the VWF level was over 150%, but the F VIII level was less 150 IU/dl, suggesting that VWF primarily contributed to venous thrombosis, independently of F VIII. Previously, the effects of VWF on platelet adhesion, aggregation, and occlusive thrombus formation were fully explained on the basis of F VIII concentrations[5]. However, a recent study of experimental thrombosis has suggested that VWF has a role in platelet adhesion, whereas F VIII is involved in subsequent thrombus growth under venous flow conditions. F VIII and VWF thus appear to be independently associated with venous thromboembolism[8]. Our observations suggest a possible association between VWF and venous thrombosis, independently of F VIII, in humans. The reason for the elevated VWF levels in our patient was unclear. Genetic factors such as blood types other than O, female sex, and black race are associated with higher VWF levels than blood group O, male sex, and white race[9,10]. Although these factors may influence VWF levels, elucidation of the cause of the elevated levels in our patient must await further future studies.

Various treatments are used to manage dural venous thrombosis, including heparin/warfarin, urokinase, sinus thrombectomy, and decompressive craniectomy. Our patient had intracranial hemorrhage, cerebral edema, and resultant infarction, associated with severe neurological symptoms despite heparin infusion peripherally. We therefore performed balloon percutaneous transluminal angioplasty and mechanical thrombectomy. These procedures for direct revascularization of the sinus successfully recanalized the intracranial sinuses, subsequently improving neurological symptoms, followed by clinically significant radiographic improvement in response to anticoagulant therapy. We believe that the local thrombolytic treatment changed the course of the patient. This combination of therapies may be a viable treatment option in patients with extensive thromboses of the dural sinuses with progressive neurological deterioration.

VWF may contribute to CSVT and that inhibition of VWF activity potentially has a role in the future treatment of pathological conditions related to venous thrombosis.

## Figures and Tables

**Figure 1:**
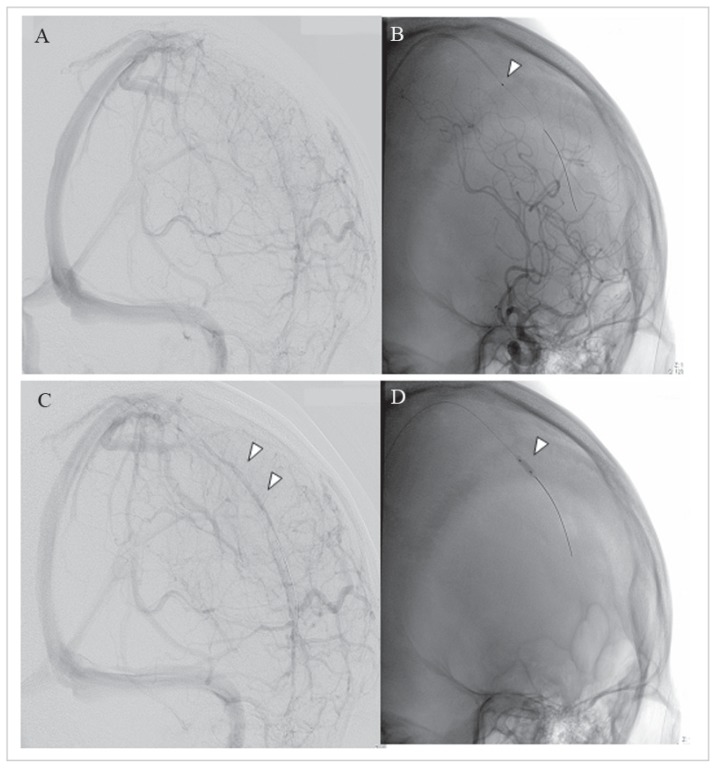
**A:** A left internal carotid angiogram (lateroposterior view, venous phase), showing severe lack of the anterior superior sagittal sinus (SSS). **B:** The Thrombuster II catheter (arrowhead indicates body of the catheter) has been advanced into the anterior portion of the SSS. **D:** The balloon catheter (white arrow) has been advanced into the anterior portion of the SSS. **C:** A left internal carotid angiogram (lateroposterior view, venous phase) after direct revascularization of the sinus, showing moderate recanalization of the SSS (white arrow).
